# Role of SAM Chain Length in Enhancing the Sensitivity of Nanopillar Modified Electrodes for Glucose Detection

**DOI:** 10.3390/s90301295

**Published:** 2009-02-26

**Authors:** Venkataramani Anandan, Rajan Gangadharan, Guigen Zhang

**Affiliations:** 1 Micro/Nano Bioengineering Lab and; 2 Faculty of Engineering, The University of Georgia; 3 Bioengineering, Electrical & Computer Engineering, Clemson University,; 4 Institute for Biological Interfaces of Engineering, Clemson University

**Keywords:** Gold nanopillar modified electrodes, self assembled monolayer, alkanethiols, electrochemical processes, glucose detection, biosensors

## Abstract

In this report, alkanethiol self assembled monolayers (SAM) with two different chain lengths were used to immobilize the functionalizing enzyme (glucose oxidase) onto gold nanopillar modified electrodes and the electrochemical processes of these functionalized electrodes in glucose detection were investigated. First, the formation of these SAMs on the nanopillar modified electrodes was characterized by the cyclic voltammetry and electrochemical impedance spectroscopy techniques, and then the detection sensitivity of these functionalized electrodes to glucose was evaluated by the amperometry technique. Results showed that the SAM of alkanethiols with a longer chain length resulted in a higher degree of surface coverage with less defect and a higher electron transfer resistance, whereas the SAM of alkanethiols with a shorter chain length gave rise to a higher detection sensitivity to glucose. This study sheds some new insight into how to enhance the sensing performance of nanopillar modified electrodes.

## 1. Introduction

For many in-vivo biomedical applications, it is desirable that biosensors are miniature devices such that they can be implanted inside the body without hindering the body’s normal physiological functions and causing any disturbing physical appearance. Miniaturized biosensors, however, can usually handle small volumes of analyte in-vivo [1–3]. To be able to generate reliable measurements, these miniature biosensors need to be highly sensitive to produce a sufficiently high signal response to a small volume of analyte.

For this purpose, improvements for the sensitivity of biosensors have been explored through incorporation of nanostructures such as nanopillars [4–6] into the electrodes of the biosensors. Since most of these nanostructures are made of inorganic materials, they have to be functionalized for biorecognition purposes [7, 8]. To functionalize these electrodes, biosensitive molecules such as enzymes need to be immobilized onto the electrode surface through the use of anchoring molecules. Therefore, the ability to improve the performance of these inorganic-based electrodes relies not only on the incorporated nanostructures but also the anchoring molecules [9].

Immobilization of enzymes onto electrode surfaces using self-assembled monolayers (SAM) of alkanethiols has been commonly used because SAM molecules offer easy formation of well ordered and stable monolayers of molecules for anchoring various biosensitive molecules [7, 10]. In an earlier work [4], we modified the conventional flat gold electrodes with standing gold nanopillars to form three dimensional (3D) electrodes (for this reason the two terms ‘nanopillar modified electrode’ and ‘3D electrode’ are used interchangeably hereafter). After functionalization with glucose oxidase (GOx) by using SAM molecules of 3-mercaptopropionic acid, these nanopillar modified electrodes exhibited a sensitivity measurement of 3.13 μA·mM^−1^·cm^−2^, which is much higher than that for a gold nanotube modified electrode (0.4 μA·mM^−1^·cm^−2^; [6]).

As we learned from the literature [9, 11], on a flat-surface electrode SAM molecules of a longer chain length (e.g., 11-mercaptoundecanoic acid, or MUA) produced a more ordered assembly of molecules with a higher degree of surface coverage and less defect than those of a shorter chain length (e.g., 3-mercaptopropionic acid, or MPA). But MPA SAM on a flat electrode exhibited a lower electron transfer resistance than MUA SAM and gave rise to higher detection sensitivity than MUA SAM. Since the surface coverage of these SAM layers mainly depends on the surface morphology of the electrodes [12], it is believed that the presence of the closely packed standing nanopillars in the 3D electrodes may alter the formation of the alkanethiol SAMs at the surface. This belief led us to pose a new question: which type of alkanethiol SAM, a short chain or a long chain, will help facilitate a better sensing performance for nanopillar modified electrodes?

In search of an answer to this question, two alkanethiol SAMs (i.e., MPA and MUA) were used in this study as the anchoring molecules for the functionalization of nanopillar modified electrodes. After SAM treatment, the electrodes were functionalized with glucose oxidase (GOx) through covalent bonds between the GOx and the respective SAMs. After that, the electrochemical property of the formed interface was characterized for assessing the quality of the SAM coverage on these electrodes by the cyclic voltammetry (CV) and electrochemical impedance spectroscopy (EIS) techniques. Finally, the sensing performance of these SAM treated and GOx functionalized nanopillar modified electrodes was evaluated for glucose detection.

## 2. Experimental

### 2.1. Reagents and Materials

3-mercaptopropionic acid (MPA: HS-(CH_2_)_2_-COOH), 11-mercaptoundecanoic acid (MUA: HS-(CH_2_)_10_-COOH), glucose oxidase (GOx; EC 1.1.3.4 from Aspergillus Niger, 100 units/mg), *N*-hydroxysuccinimide (NHS), 2-(N-morpholino)ethanesulfonic acid (MES) and 1-ethyl-3-(3-dimethylaminopropyl) carbodiimide (EDC) were purchased from Sigma (St. Louis, MO). Porous anodic alumina templates were purchased from Whatman Inc. (Maidstone, England). Ethanol (200 Proof-absolute, anhydrous) was purchased from Pharmco Inc. (Brookfield, CT). Other reagents of analytical grade were used without further purification and all solutions were prepared with de-ionized (DI) water.

### 2.2. Preparation of Gold Nanopillar Modified Electrodes

Gold nanopillar modified electrodes were fabricated using the template method previously developed [13]. In brief, a 150 nm thick gold film was first deposited onto one side of a porous anodic aluminum (PAA) disc by sputter coating. A thicker gold layer was then electrodeposited on top of the sputtered gold film to form a strong supporting base in an Orotemp24 gold plating solution (Technic Inc, Cranston, RI) under a current density of 5 mA/cm^2^ for two minutes in a three–electrode cell (SASI VC-2, Bioanalytical Systems, West Lafayette, IN) with a platinum (Pt) counter electrode and an Ag/AgCl reference electrode. This supporting base was then masked using Miccrostop solution (Pyramid plastics Inc., Hope, AK) for insulation. After that, gold nanopillars were electrodeposited through the open pores of the PAA disc from the uncoated side under an electrical current density of 5 mA/cm^2^ at 65°C for 20 minutes. The PAA template was finally dissolved by immersing the specimen in 2M NaOH solution for 30 minutes. This procedure resulted in 3D structures having arrays of gold nanopillars standing on gold support bases. These 3D structures were cut into small pieces and used as electrodes for further studies (note that all 3D electrodes tested in this study have an exposed geometric area of 0.04 cm^2^).

Prior to SAM formation, the surfaces of these 3D electrodes were cleaned electrochemically by performing CV within a potential range from −0.5 to 1.5 V at a scan rate of 100 mV/s in 0.3M H_2_SO_4_ solution. The cleaning cycles continued until a reproducible voltammogram was obtained. After that, the electrodes were rinsed with DI water and dried with nitrogen blow.

### 2.3. SAM Formation and Characterization

For SAM formation, 3D electrodes were placed in ethanol solution containing 10 mM of either the MPA (for 6 hours) or MUA (for 24 hours) molecules for reaching saturated SAM adsorption. After washing in ethanol solution, SAM formation on these electrodes was characterized by the CV and EIS techniques at 25°C in the three-electrode cell. The CV measurements were performed by scanning the potential from −0.2 V to 0.6 V at a scan rate of 100 mV/s and the EIS measurements were performed in a frequency range from 0.1 Hz to 100 KHz with an AC signal of an amplitude of 10mV (vs. Ag/AgCl) in 0.1 M phosphate buffered solution (PBS, pH7) containing 2 mM Fe(CN)_6_^3−/4−^ (ferri:ferro=1:1) mixture as the redox probe. These electrochemical experiments were performed using a potentiostat (Solartron 1480, Houston, TX) and an impedance analyzer (Solartron 1260, Houston, TX). Prior to each test run, the electrolytic solution was bubbled with nitrogen for about 20 minutes to get rid of any dissolved oxygen, and during the test the electrolyte was blanketed with nitrogen. The obtained impedance spectra were analyzed through statistical curve-fit with an equivalent Randles circuit using ZVIEW (Scribner Associates Inc, Southern Pines, NC).

For assessing the percent defect in the SAM molecules, the voltammetric reduction peak associated with the uncovered area (i.e., the exposed gold oxide) of the SAM treated 3D electrode surface was evaluated. The ratio of the uncovered area of a SAM treated 3D electrode to that of a bare 3D electrode was calculated and the percent defect in the SAM structure determined. For these evaluations, CV measurements were obtained in 0.1 M H_2_SO_4_ by scanning the potential from −0.5 V to 1.5 V at a scan rate of 100 mV/s.

For quantifying the surface coverage (Γ) of the SAM molecules, the method reported in the literature [9, 14, 15] was used to evaluate the voltammetric reduction peak associated with SAM desorption. To do that, CV measurements were made in 0.1 M NaOH within a voltage range from −1.6 V to −0.2 V at a scan rate of 100 mV/s. From the reduction peak, the amount of charge was determined by first integrating the reduction current under the peak over time and then offsetting the value by that of a bare 3D electrode. With the formula Γ=Q/nFA [14], in which Q is the amount of charge, n (=1) is the number of electrons involved in the reaction, F (=96485 C/mol) is the Faraday constant and A (=0.04 cm^2^) is the electroactive surface area, the surface coverage of SAM molecules was determined.

### 2.4. Immobilization of GOx

After SAM treatment, the surfaces of these nanopillar modified electrodes were functionalized with glucose oxidase (GOx). To do that, the carboxyl group in the formed SAMs was activated in a freshly prepared solution of 0.1 M MES acid containing 75 mM EDC and 15 mM NHS buffered at pH 4.5 for 2 hours. After washing in 0.1M PBS the electrodes were placed in 0.1 M PBS containing 1 mg/mL of the GOx with constant stirring for 2 hours. The obtained GOx functionalized electrodes were washed thoroughly with 0.1M PBS and stored at 4°C in 0.1M PBS solution at pH 7.0 prior to testing.

### 2.5. Glucose Detection

**Figure 1.**
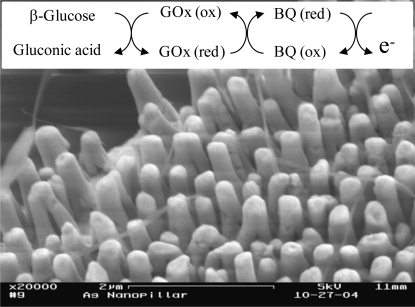
An SEM image of a typical nanopillar array modified 3D electrode. A scheme showing a cascade of events in a mediator-based glucose biosensor is shown in the inset.

For evaluating the sensing performance of these SAM treated and GOx-functionalized nanopillar modified electrodes, the amperometric currents of these 3D electrodes in response to glucose at various concentrations were measured. For the electrode reactions, when the GOx functionalized electrodes are placed in a solution containing glucose, glucose will first react with GOx to form gluconic acid and reduced-GOx. The reduced-GOx will then be converted back to its original form by reacting with p-benzoquinone, a mediator having better solubility than most other popular mediators [16]. In this reaction, the mediator gets reduced and then converted back to its original state at the electrode surface by giving away electrons. A cascade of reactions is shown schematically in the inset in figure 1. Amperometric measurements were made in 0.1M PBS (20 ml) containing 3mM p-benzoquinone by adding various amounts of 1M glucose using the three-electrode electrochemical cell. For all tests, a constant potential of 0.35 V was applied to the electrodes and the solution was stirred continuously for ensuring an instant equilibrium for mass transport. During each test run, the background current was allowed to stabilize before a drop (50 μL) of 1M glucose was added to the solution, and after the amperometric current response reached a steady-state, another drop of glucose was added and the corresponding current response was measured until a new steady state was reached. In this manner, each incremental drop of glucose to the solution caused an equivalent increase in glucose concentration of 2.5 mM approximately.

## 3. Results and Discussion

### 3.1 Characterization of the Nanopillar Modified Electrodes

Figure 1 shows an SEM image of a typical nanopillar modified electrode. From this image, the diameter of the nanopillars is estimated to be around 200 nm and the height of the nanopillars around 2.5 μm. The surface area of the 3D electrodes was characterized by a roughness factor which was determined from the electrochemical cleaning CV curves using the method described in our previous report [4]. The roughness factor for the 3D electrodes tested in this study is approximately 45.

### 3.2. Characterization of SAM Formation

**Figure. 2.**
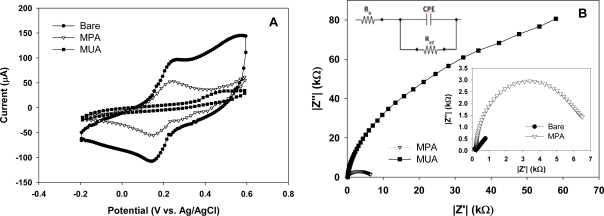
(A) CV curves obtained for a bare, a MPA and a MUA treated electrodes evaluated with Fe(CN)_6_^3−/4−^ as the redox couple. (B) The corresponding Nyquist plots from the impedance measurements for the same electrodes with a close-up view of the low impedance range given in the lower inset. A Randles equivalent circuit consisting of a solution resistance (Rs), an electron-transfer resistance (Ret) and a constant phase element (CPE) capacitor is given in the upper inset.

In figure 2A the CV curves obtained for a bare, a MPA and a MUA treated 3D electrodes evaluated in the presence of the redox couple are shown. In comparison between the bare and SAM treated electrodes, the bare one exhibits much higher redox peak currents. Between the two SAM treated electrodes, the MUA treated electrode exhibits lower redox peak currents than the MPA treated electrode, suggesting a higher degree of blockage for electron transfer resulting from MUA molecules than from MPA molecules. For both the bare and MPA treated electrodes the CV curves show a reversible redox event at the electrode surface with the electron transfer limited by diffusional mass transport. By contrast, the CV curves for the MUA treated electrode exhibits highly irreversible redox behavior, confirming a high degree of blockage at the electrode surface for electron transfer. Taken together, the above results indicate that both MUA and MPA molecules form SAM structures covering the electrode surface and that there are more MUA molecules than MPA molecules blocking the pathways for electron transfer across the electrode-electrolyte interface for facilitating redox reactions, owing possibly to the longer chain length of MUA molecules forming more lateral molecular bonds.

Figure 2B shows the corresponding impedance spectra (Nyquist plots) for these 3D electrodes. The two SAM treated electrodes show semicircular Nyquist plots whereas the bare electrode exhibits a straight line plot (see the lower inset plot in Fig. 2B). Since a semicircular feature is indicative of blockage for electron transfer across the electrode/electrolyte interface, this result confirms the formation of SAM molecules on the electrode surfaces. Moreover, the MUA treated electrode exhibits a larger semicircle than the MPA treated electrode, suggesting a high degree of SAM coverage for MUA than for MPA molecules. To put this in a quantitative sense, a Randles equivalent circuit (see the upper inset in Fig. 2B) consisting of a solution resistance (R_s_), an electron-transfer resistance (R_et_) and a constant phase element (CPE) capacitor was used to fit the obtained semicircular Nyquist plots to resolve the values for R_et_. Note that a detailed discussion of the equivalent circuit and the relevant parameters can be found in our previous work [7]. As listed in table 1, the R_et_ value obtained for the MUA treated electrode is much higher (about 33 times) than that for the MPA treated electrode, thus confirming that MUA molecules indeed post a higher electron transfer resistance at the electrode surface than MPA molecules.

To further confirm the immobilization of GOx onto the electrode surface, we also measured the impedance of the MPA covered electrode after enzyme immobilization. From this we found that the electron transfer resistance (R_et_) increased by 3.96 times due to enzyme immobilization for the MPA covered electrode. This increase is consistent with the result reported for a flat surface [17]. However, to use this ratio to quantify the amount of immobilized GOx, we would need to have the ratio date for the MUA covered electrode, which was not obtained. A systemic study for quantifying the amount of enzyme is underway and results will be reported separately.

**Figure 3.**
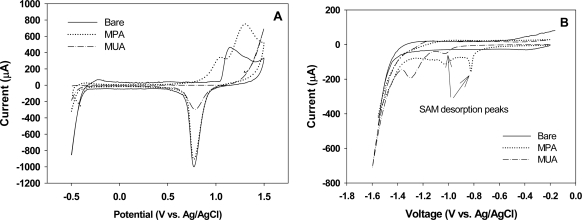
(A) CV curves obtained for the bare and MPA and MUA treated 3D electrodes in quantifying the percent defect in SAM molecules in electrolyte containing 0.1 M H_2_SO_4_. (B) CV curves obtained for the bare and MPA and MUA treated 3D electrodes in evaluating SAM desorption in electrolyte containing 0.1M NaOH.

The CV curves obtained from the gold-oxide reduction experiments performed in H_2_SO_4_ are shown in figure 3A. All these CV curves exhibit an Au-oxide reduction peak at around 0.78 V, indicating that all these 3D electrodes possess a certain amount of defect, or the exposed gold oxide, on the SAM treated electrode surfaces. By the ratio of the area under the reduction peak (by integrating the CV curve under the peak) of the SAM treated 3D electrode to that of the bare 3D electrode, a measure of the percent defect in these SAMs was obtained (see table 1): the percent defect is approximately 87.3% and 37.8% for the MPA and MUA covered electrodes, respectively. These values are high when compared with flat electrodes: 52% for the MPA and 0% for the MUA cases [11].

Figure 3B shows the CV curves obtained for evaluating the voltammetric reduction peak associated with desorption of MPA and MUA molecules. From the CV curves, two peak currents are visible for both the MPA and MUA treated 3D electrodes. We believe that the peak current at around −0.82 V for MPA and around −1.03 V for MUA is due to the cleavage of the gold-sulfur bond. This observation is consistent with the reported results in literature. For example, a peak desorption current between −0.6 to −0.9 V was found for short alkanethiols (n=2 to 6) and between −1.0 to −1.2 V for long alkanthiols (n=11 to 18) [9, 15, 18, 19]. The nature of the second peak at a higher reduction potential in both curves is not known at this time, although it was also observed by Imabayashi et al. with flat surfaces [18]. Based on these desorption peak currents, the desorption charge was determined by integrating the reduction peak from −0.8 V to −0.9 V for the MPA treated electrode and from −1.0 V to −1.2 V for the MUA treated electrode. The values for Γ were then calculated for the MUA and MPA treated electrodes as listed in table 1. Comparing with the reported values for the surface coverage of MPA (5.12×10^−10^ mol/cm^2^) and MUA (8.30×10^−10^ mol/cm^2^) SAMs on flat surfaces [11], the values for nanopillar modified electrodes are roughly 27 and 28 times higher respectively. This increase can be attributed to the increase in the electroactive surface area in the 3D electrodes. This increase, however, does not correspond to the actual increase in the surface area of 45 times. This phenomenon may be attributed to the high percent defect in the SAM structures on the nanopillar modified electrodes as well as the presence of rough surfaces at the top end of the nanopillars as seen in the SEM image. A similar observation for electrodes of rough surfaces was made by others where they attributed it to the presence of a large number of edges in the rough surfaces leading to more defect in the SAM structures [20,21].

### 3.3. Amperometric Responses

**Figure 4.**
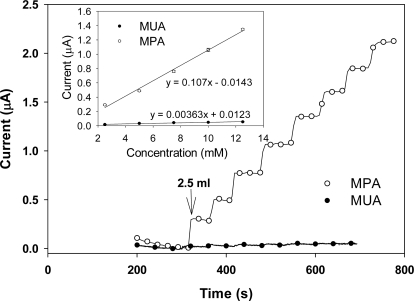
Amperometric current measurements obtained for the MPA and MUA treated 3D electrodes in response to glucose at various concentrations. The inset shows the two linear calibration curves.

Figure 4 shows the strip-chart measurements of the amperometric current responses of the SAM treated and GOx functionalized 3D electrodes taken as drops of glucose were added. In general, the current level for the MPA treated electrodes is much higher than the MUA treated electrodes. For calibrating detection sensitivity, the steady-state current at each glucose concentration was first taken and plotted against the corresponding cumulative glucose concentration. Then the detection sensitivity was determined by the slope of each calibration plot (evaluated through a linear regression analysis) normalized by the geometric area of the corresponding electrode. The inset in figure 4 shows the variation of the amperometric steady-state current with glucose concentration for the two electrodes. The detection sensitivity is found to be 2.68 μA·mM^−1^·cm^−2^ and 0.09 μA·mM^−1^·cm^−2^ for the MPA and the MUA treated 3D electrodes, respectively. The sensitivity value for the MPA treated 3D electrode (2.68 μA·mM^−1^·cm^−2^) is slightly lower than the one obtained in our earlier work (3.13 μA·mM^−1^·cm^−2^; [4]), which is as expected because the electrodes tested here had a roughness factor of 45 whereas the electrodes tested earlier had a roughness factor of 60.

The results presented above indicate that the two alkanethiol SAMs (i.e., MPA and MUA) have led to a similar trend in terms of SAM surface coverage, percent defect, electron transfer resistance and glucose detection sensitivity for both the nanopillar modified electrodes and flat electrodes, albeit the exact values are different. This similarity can be attributed to the fact that although the presence of these closely packed standing nanopillars affects the SAM formation, the gap spacing between these nanopillars is still relatively large in comparison with the chain length of MPA and MUA molecules. Under this circumstance, the distance between the electrode surface and the redox center (where the GOx catalyzed glucose oxidation occurs) which is controlled by the chain length of the SAM molecules will play a dominating role in dictating the sensing performance of these SAM treated and GOx functionalized 3D electrodes. This argument is supported by the fact that higher detection sensitivity was observed for the MPA treated electrodes than for the MUA treated ones. As a side note of confirmation, we also tested 16-mercaptohexadecanoic acid (MHA: HS-(CH_2_)_15_-COOH, Sigma, St. Louis, MO) SAM and found that the MHA treated 3D electrodes exhibited an electron transfer resistance 1.3-fold higher than that of MUA treated ones and they did not show any amperometric current response to glucose.

To put the sensitivity values of the 3D electrodes in perspective with reference to those of flat electrodes for glucose detection, the same glucose detection studies were performed using a flat gold disc electrode with a circular area of 0.02 cm^2^ (Bioanalytical Systems, West Lafayette, IN). Prior to and in between test runs, the disc electrode was polished first with a 1 μm diamond polishing sheet and then with a 50 nm alumina polishing sheet and rinsed in DI water. For the MPA and MUA treated flat electrodes, the sensitivity values were found to be 0.47 μA·mM^−1^·cm^−2^ and 0.052 μA·mM^−1^·cm^−2^, respectively. For the MPA treated electrodes, the presence of the nanopillars caused a 6-fold increase in detection sensitivity. Although a 6-fold increase is high, it is not higher enough with respect to the 45-fold increase in the surface area of these 3D electrodes. This disparity is certainly related to the increased amount of blockage for electron transfer from the SAM molecules covering the 3D electrode surface, but it may also suggest that the amount of GOx functionalized onto the 3D electrodes is not proportional to the available SAM surface as in the case with flat electrodes.

## 4. Conclusions

From this study we observed the same general trend in terms of SAM surface coverage, percent defect, electron transfer resistance, and glucose detection sensitivity when using MPA and MUA as the anchoring SAMs for the functionalization of nanopillar modified electrodes and flat electrodes. For nanopillar modified electrodes tested here, the longer MUA SAM produced a higher electron transfer resistance and lower percent defect than the shorter MPA SAM, but the shorter MPA SAM led to higher sensitivity in glucose detection than the longer MUA SAM.

## Figures and Tables

**Table. 1.** Left: the resolved R_s_ and R_et_ values based on the Randles circuit (fitting errors given in parenthesis). Right: the obtained values for the surface coverage and percent defect.

Electrodes	R_s_ (ohm)	R_et_ (ohm)	SAM	Γ (10^−8^ mol·cm^−2^)	% defect	% adsorption
Bare	227.0 (2.0%)	589.5 (5.0%)	MPA	1.38±0.1	87.3	12.7
MPA	256.6 (0.9%)	6281.0 (1.7%)	MUA	2.37±0.3	37.8	62.2
MUA	229 (1.0%)	209370 (4.3%)				
